# The role of podocyte injury in the pathogenesis of Fabry disease nephropathy

**DOI:** 10.1590/2175-8239-JBN-2024-0035en

**Published:** 2024-07-19

**Authors:** José Tiburcio do Monte, Gianna Mastroianni Kirsztajn

**Affiliations:** 1 Universidade Federal do Piauí, Departamento de Clínica Geral, Teresina, PI, Brazil.; 2 Universidade Federal de São Paulo, Departamento de Medicina, São Paulo, SP, Brazil.

**Keywords:** Fabry Disease, Podocyte, Glomerular Filtration Barrier, Autophagy, Renal Insufficiency, Chronic

## Abstract

Renal involvement is one of the most severe morbidities of Fabry disease (FD), a multisystemic lysosomal storage disease with an X-linked inheritance pattern. It results from pathogenic variants in the GLA gene (Xq22.2), which encodes the production of alpha-galactosidase A (α-Gal), responsible for glycosphingolipid metabolism. Insufficient activity of this lysosomal enzyme generates deposits of unprocessed intermediate substrates, especially globotriaosylceramide (Gb3) and derivatives, triggering cellular injury and subsequently, multiple organ dysfunction, including chronic nephropathy. Kidney injury in FD is classically attributed to Gb3 deposits in renal cells, with podocytes being the main target of the pathological process, in which structural and functional alterations are established early and severely. This configures a typical hereditary metabolic podocytopathy, whose clinical manifestations are proteinuria and progressive renal failure. Although late clinical outcomes and morphological changes are well established in this nephropathy, the molecular mechanisms that trigger and accelerate podocyte injury have not yet been fully elucidated. Podocytes are highly specialized and differentiated cells that cover the outer surface of glomerular capillaries, playing a crucial role in preserving the structure and function of the glomerular filtration barrier. They are frequent targets of injury in many nephropathies. Furthermore, dysfunction and depletion of glomerular podocytes are essential events implicated in the pathogenesis of chronic kidney disease progression. We will review the biology of podocytes and their crucial role in regulating the glomerular filtration barrier, analyzing the main pathogenic pathways involved in podocyte injury, especially related to FD nephropathy.

## Introduction

Fabry disease (FD) is a genetic lysosomal storage disorder with an *X*-linked inheritance pattern, with an incidence of approximately 1 in 40-60,000 live-born males^1^. The disease results from pathogenic variants of the GLA gene (located in the Xq22.2 region), responsible for transcribing the alpha-galactosidase A (α-Gal) enzyme, which acts in the catabolism of glycosphingolipids. The failure of this enzyme’s activity produces a progressive accumulation of toxic intermediate substrates, such as globotriaosylceramide (Gb3), within lysosomes, triggering a slow process of cellular injury that later manifests as dysfunction in target organs such as the heart, nervous system, and kidneys^2^.

More than a thousand mutations in the GLA gene have been described to date, correlated with the heterogeneous and multisystemic presentation of FD, encompassing a broad spectrum of clinical manifestations. These include the classic phenotype in males and late-onset disease. Affected women, being heterozygous (XX), typically present with attenuated clinical manifestations due to the phenomenon of random inactivation of one of the X chromosomes at the embryonic stage^3^.

Renal involvement is the leading cause of morbidity and mortality in FD. Nephropathy in this disorder represents a podocytopathy of genetic and metabolic origin, as podocyte injury plays a central role in the pathogenesis^4–6^. In the overall context, podocyte injury and its consequences are events present in most proteinuric nephropathies and are furthermore implicated in the progression of chronic kidney disease (CKD) of various etiologies^7^.

FD nephropathy is complex and involves multiple molecular aspects and structural alterations that precede clinical events^8–10^. The metabolic derangement induced by GLA gene inactivation seems to promote the production of secondary mediators that culminate in podocyte injury, triggering chronic progressive nephropathy, which results in glomerulosclerosis and kidney failure. It is necessary to deepen our understanding of the molecular mechanisms involved in FD nephropathy from the genetic defect, going beyond the lysosomal deposits of Gb3^8^. The cumulative storage of substrates, caused by enzyme deficiency, may highlight only one aspect of the disease pathogenesis, while the involved molecular mechanisms will only be fully understood if we consider the affected cellular functions^9,11^.

In this review, we will explore the biology of podocytes and their role in regulating the glomerular filtration barrier, investigating the sequence of molecular and cellular events implicated in the pathogenesis and progression of FD nephropathy. Although rare, addressing this genetic disorder could contribute to understanding the pathological processes shared by the most prevalent nephropathies.

### Glomerulus and Glomerular Filtration Barrier

Blood filtration performed in the renal cortex by the glomeruli is essential for regulating the volume and composition of the organism, maintaining homeostasis and ensuring the integral performance of renal function. The glomeruli form a spherical structure delimited by Bowman’s capsule. They consist of a “coil-shaped” capillary network of high permeability, subjected to elevated hydrostatic pressure established by the difference in resistance between the efferent and afferent arterioles, responsible for outflow and inflow of blood into the glomerulus, respectively^12^.

Plasma glomerular ultrafiltrate is the largest fluid transfer in the body, with a volume of approximately 180 liters per day. In this process, continuously performed by healthy glomeruli at a rate of 100–125 mL/min/1.73 m^2^ of body surface area, it is necessary to preserve within the capillaries the essential components of blood: cells, nutrients, and high molecular weight proteins such as albumin. Meanwhile, electrolytes, uremic toxins, water, glucose, and small solutes are released into the urinary space of Bowman’s capsule to be processed by the renal tubules in the medullary region, ultimately forming urine^13^.

The low urinary excretion of albumin (which physiologically does not exceed 30 mg in 24-hour urine), given its high concentration in the blood (exceeding 4000 mg in 100 mL of plasma), indicates the highly selective property of the glomerulus (greater than 99.99%) in preserving this protein^14^.

The glomerular filter is centered on the glomerular filtration barrier (GFB), a structure consisting of three parallel layers: (i) internally, the fenestrated endothelium, (ii) in the center, the glomerular basement membrane (GBM), and (iii) externally, the layer of visceral epithelial cells or podocytes^15^. GBM is formed by the fusion of the extracellular matrix produced by the underlying endothelium and the overlying podocytes, which structurally forms a network. The main constituent of this network is type IV collagen, to which glycoproteins (heparan sulphate, laminin and proteoglycans) bind, expressing a negative electrical charge. They impart electrostatic properties, which help prevent the filtration of anionic proteins, such as albumin^16^.

The GFB therefore acts as a highly efficient selective filter, expressing mechanical and electrostatic barriers that simultaneously prevent the passage of molecules based on size, shape, and charge. This process produces the ultrafiltrate in flow and composition, on which the functional performance of the kidney depends^16^.

### Structure and Function of Podocytes

The outer layer of the GFB is strategically composed of podocytes, visceral epithelial cells that surround the glomerular capillaries from the vascular pole, delimiting the boundary between vascular structures and the urinary space^17^. A healthy glomerulus contains approximately 500 to 600 podocytes, representing 30% of its cellular components^18^.

Podocytes are the most differentiated and specialized cells in the kidney^19^. They consist of a large cell body that floats in the urinary space, from which emerges an extensive, arborized cytoplasm that projects towards the capillaries, forming the primary and secondary processes, and the pedicels (foot processes)^17^. The pedicels cover the entire length of the GBM, forming interdigitations between themselves and with the pedicels of adjacent podocytes, through specialized intercellular junctions called slit diaphragms.

**Figure 1:**
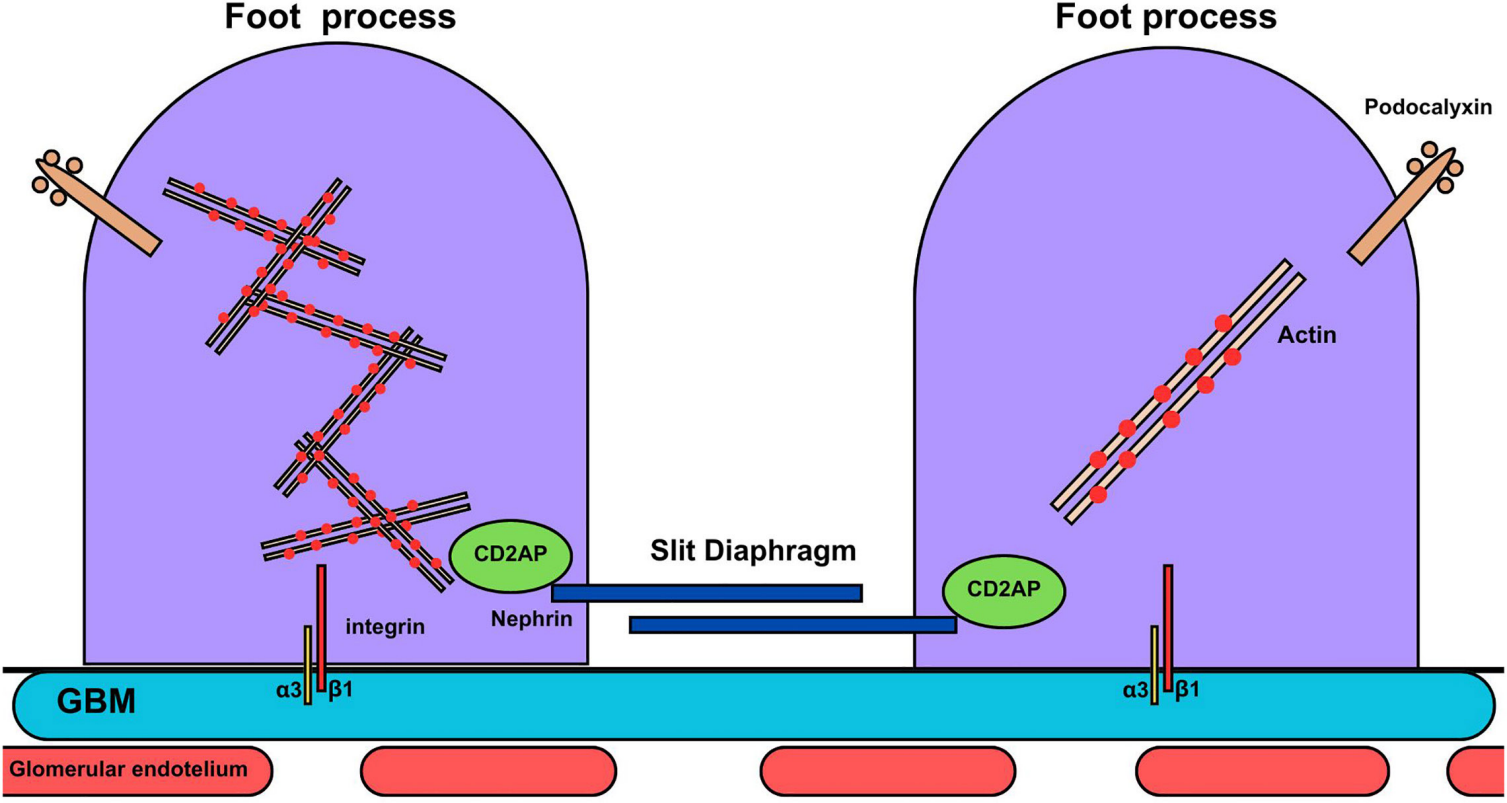
Diagram representing podocyte pedicels interconnected in the slit diaphragm adhered to the GBM and the underlying endothelium, forming the GFB. Highlighting the main structural proteins in their corresponding domains: nephrin (junctional domain of the slit diaphragm), podocalyxin (apical domain) and integrins α3β1 (basal domain), articulated to the actin cytoskeleton.

The complex molecular architecture of podocytes includes several interconnected proteins, grouped into three main domains in the pedicels: basal, apical and junctional (Figure 1)^17^. The basal domain represents the contact surface adhered to the GBM, containing different types of integrins and dystroglycans, with α3β1 integrin being the most abundant and primarily responsible for the focal adhesion of the podocyte to the GBM, preventing its detachment from the glomerulus. The apical domain, facing the urinary space, has an anionic surface charge, conferred by the presence of proteins such as podocalyxin. In addition to reinforcing the electrostatic barrier of the GFB, hindering albumin from escaping, these proteins prevent podocyte adhesion to Bowman’s capsule, keeping adjacent pedicels separated. The junctional domain encompasses the slit diaphragm, whose zipper-like interdigitating pattern establishes the last barrier crossed by the glomerular filtrate (Figure 1)^20^.

The slit diaphragm symbolizes the functional unit of the podocyte by exerting the narrowest and most definitive barrier responsible for the selective permeability of the glomerulus^20,21^. This structure is filled with specific transmembrane proteins such as nephrin, the main component of this specialized intercellular junction, essential for the organization and maintenance of podocyte integrity (Figure 1)^22^. Actin microfilaments form the cytoskeleton of pedicels, providing structural support, as well as allowing for contraction and relaxation of podocytes, regulating the filtration surface of glomerular capillaries. Other proteins are essential, such as the adapter protein CD2AP (CD2-associated protein) and podocin, acting as anchors linked to nephrin, connecting the slit diaphragm to the actin cytoskeleton (Figure 1). The slit diaphragm protein complex and the nephrin-CD2AP interaction are essential for the selective permeability and ultrafiltration of plasma to be rigorously performed by the GFB^22,23^.

Thus, the molecular architecture of pedicels configures a complex network of interactions between different proteins and signaling molecules, ensuring precise communication between different cellular compartments and the adjacent environment through specific transmembrane proteins. These proteins act as receptors linked to the actin cytoskeleton, modulating the shape and function of the podocytes, providing rapid and effective responses to changes in the glomerular environment^21^.

In addition to controlling the surface and permeability of the GFB, podocytes have other functions. These include synthesizing and repairing GBM components, producing paracrine substances that act as growth factors on endothelial and mesangial cells, such as VEGF (vascular endothelial growth factor) and PDGF (platelet-derived growth factor), promoting intraglomerular communication through multiple signaling pathways. Podocytes also remove unfiltered proteins and immunoglobulins by endocytosis, preventing obstruction of the filtration membrane. They also interact with the immune system, acting as antigen-presenting cells or as receptors for components of the complement system and immunoglobulins, and may be targeted by immune-mediated insults in some glomerulopathies^24^.

It is thus understood that renal function and integrity of the GFB are completely dependent on the quantity and proper functioning of podocytes. All this extraordinary work, encompassing the connection and monitoring of other glomerular cells, places the podocyte in a commanding position within the glomerulus.

## Fabry Disease Nephropathy

**Figure 2:**
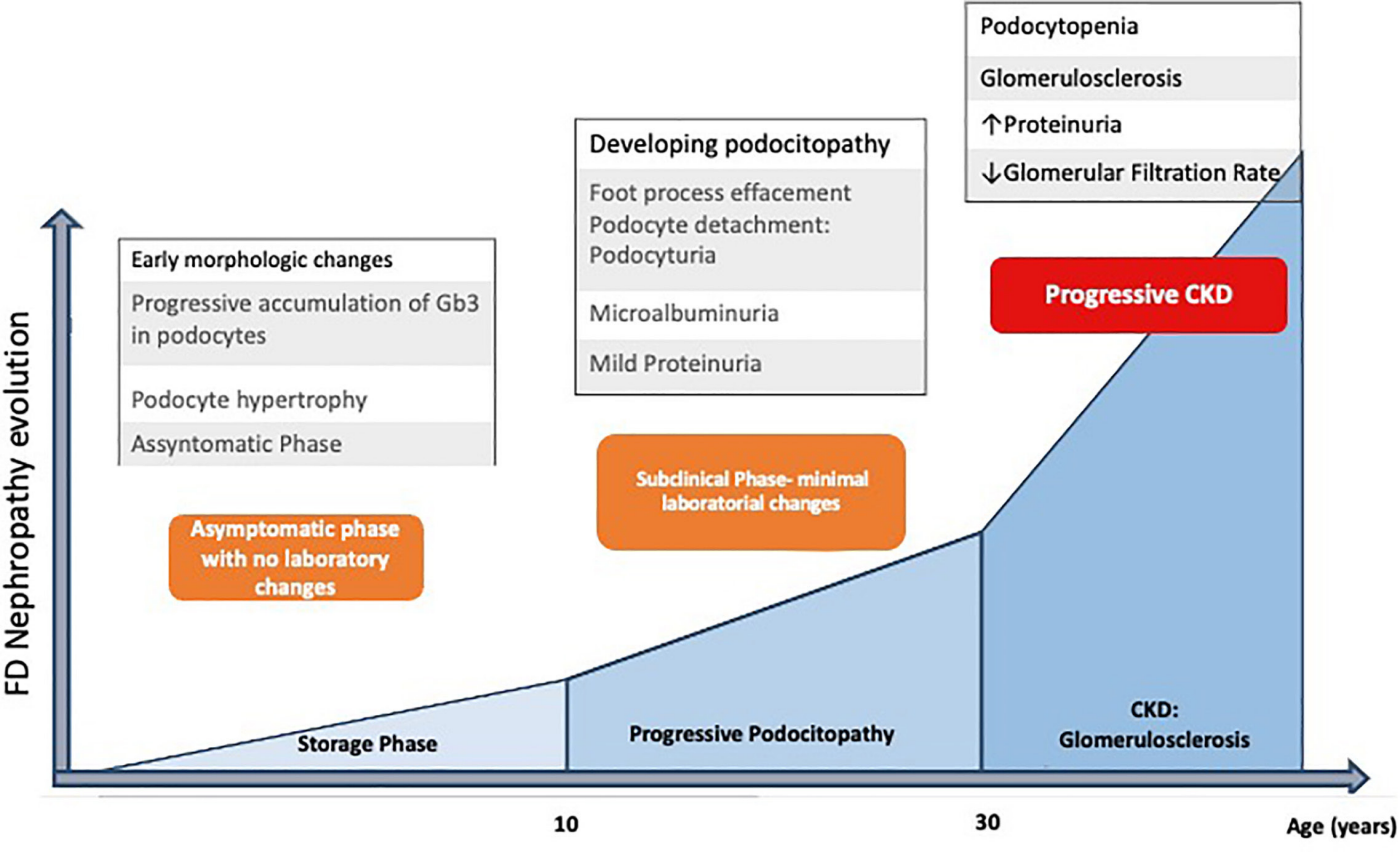
Summary of the evolution of Fabry disease nephropathy in men with the classic phenotype of the disease, correlating clinical and laboratory picture with the corresponding morphological changes.

Renal impairment represents a major cause of morbidity and mortality in FD^25,26^. In the classic phenotype of the disease, most male carriers of *nonsense* mutations develop microalbuminuria in childhood and adolescence, progressing to clinical proteinuria from youth onwards. Between the third and fourth decades of life, chronic kidney disease (CKD) progression begins, reaching its most advanced stage around the fifth decade of life, thus requiring the introduction of renal replacement therapy (dialysis or transplantation) (Figure 2)^6,25,27,28^. In women and individuals with late-onset variants, renal impairment tends to be less severe and have a milder course.

From a clinical standpoint, renal involvement in FD is characterized by proteinuria of increasing intensity and a gradual decline in renal function. Proteinuria may emerge in childhood or adolescence, reflecting the first clinical manifestation of this nephropathy^29^. However, kidney biopsy analyses have revealed prominent ultrastructural morphological changes in podocytes before the clinical signs of kidney disease become evident^30^. The renal histopathology of FD highlights hypertrophic podocytes with cytoplasmic vacuolization of a foamy appearance and multilamellar inclusion bodies, described as myelin figures or zebra bodies, corresponding to Gb3 deposits accumulated in lysosomes^31^.

In the natural history of the classic phenotype of FD nephropathy, Gb3 deposits are already early present in podocytes, beginning in intrauterine life^32^. Over time and the patient’s aging, a greater proportion of the cytoplasm is occupied by unprocessed deposits, impairing functionality^30,33^. The subclinical stage, observed in childhood and adolescence and characterized by microalbuminuria, corresponds to morphological changes such as foot process effacement and podocyte hypertrophy. In the subsequent stage, the worsening of podocyte morphological parameters is associated with increased proteinuria and worsened renal function, establishing the clinical phase of this nephropathy (Figure 2)^34^. CKD then progresses rapidly, reflecting the accelerated depletion of podocyte numbers (podocytopenia), and the development of glomerulosclerosis, intensified from the age of 30 (Figure 2)^27,28,33^. The severity of podocyte injury is thus strongly associated with the progression of nephropathy in FD (Figure 2).

In this context, renal function tests routinely used in clinical practice, which identify proteinuria and reduced glomerular filtration rate, already reveal advanced kidney injury^35^. In a previous study, we identified a positive correlation between podocyturia and urinary albumin excretion in FD patients^36^. However, podocyturia, which could play the role of an early biomarker for renal involvement, occurs discontinuously and variably, and is not available in routine laboratory tests.

These data reinforce the need for research into new biomarkers of podocyte injury that enable identification of the nephropathy stage preceding the onset of irreversible structural and functional alterations, thus optimizing the indication and efficacy of specific treatments, such as enzyme replacement therapy or pharmacological chaperones (migalastat)^5,33,37^.

### Pathophysiology of Podocyte Injury in Fabry Disease Nephropathy

Podocyte injury is the determining factor in the development and progression of FD nephropathy. Gb3 lysosomal deposits constitute the first stage of complex pathological pathways that result in podocyte injury in this disease^8,30^.

Since podocytes exhibit limited regenerative capacity, characteristic of post-mitotic cells, podocyte injury and loss, whether due to apoptosis or detachment, are irreversible events. Podocytes are injured both at the molecular and morphological levels in the FD pathological process.

#### Morphological changes

Having reached a high stage of differentiation and specialization, typical of post-mitotic cells, podocytes have lost their ability to divide, making them particularly vulnerable to various insults^38^. Podocytes rarely undergo mitosis, and when they enter the cell cycle, they do not complete cytokinesis, resulting in catastrophic (aberrant) mitosis. In this case, defective cell division produces polyploid or multinucleated cells without adherence to the GBM^39^.

In response to injury, adaptive morphological changes occur in the podocytes, such as pedicel effacement, hypertrophy, and detachment. The severity of these responses depends especially on the intensity and duration of the insult, which in FD is continuous due to the cumulative stock of pathogenic deposits that intensify with the patient’s advancing age.

**1) Fusion and effacement of pedicels**

Pedicel effacement is characterized by the loss of typical interdigitations, resulting from the fusion and disappearance of the slit diaphragm, with flattening of the podocyte layer^39,40^. This process is triggered by disruption of the actin cytoskeleton, and was first visualized in the 1950s with the advent of electron microscopy. This finding is associated with proteinuric glomerulopathies, regardless of etiology^40^.

Podocyte injury, due to the progressive accumulation of Gb3, is associated with foot process effacement. This morphological alteration is considered an early biomarker of the developing nephropathy, and has been described in children aged 10 and over with the classic FD phenotype, who had not yet shown clinical evidence of renal impairment (Figure 2)^10,32,41^. The density of Gb3 inclusions in podocytes and the fusion of pedicels become more pronounced with the patient’s age, correlating with increased proteinuria^30,33^.

**2) Podocyte detachment and podocyturia**

Chronic and persistent podocyte injury leads to their detachment from the glomerulus, due to either increased mechanical tension and/or failure to adhere to the GBM^42^. Hydrostatic pressure, although necessary for filtration, generates hemodynamic stress on podocytes when it is increased beyond physiological levels. Excessive biomechanical stress caused by glomerular hyperfiltration, as observed in renin-angiotensin system hyperactivity, increases the shear stress exerted by the glomerular ultrafiltrate on pedicels, opposing their attachment to the glomerular capillary^43,44^. Adherence to impaired GBM plays a major role, especially in genetic or acquired conditions involving molecular alterations in the structural proteins of the slit diaphragm, leading to the appearance of significant podocyturia^20^. Furthermore, the intensity of podocyturia has been correlated with the severity of nephropathy in FD^42^.

**3) Podocyte hypertrophy**

In FD, podocytes exhibit increased volume and hypertrophy due to the continuous and cumulative Gb3 deposition^33^. Moreover, the reduction in the number of podocytes per glomerulus, due to excessive detachment, stimulates remodeling of the remaining cells to cover the gaps in the podocyte cover of the GBM^39,45,46^. Glomerular volume increases rapidly with age, while podocyte numbers decrease, resulting in reduced podocyte density^33^. Compensatory podocyte hypertrophy weakens their adhesion to GBM, rendering them more vulnerable to injury, worsening dysfunction, and accelerating the progression of chronic kidney disease^39,47,48^.

Additionally, in the event that compensatory hypertrophy is unable to compensate for podocyte loss, synechiae develop, and capillary loops collapse, triggering the process of glomerular sclerosis^7^.

While changes such as pedicel effacement or hypertrophy are potentially reversible, the loss of podocytes through detachment or apoptosis represents an irreversible event^39^. At this stage, podocyte injury leads to a reduction in the number and density of these cells in the glomerulus, culminating in the irreversible process of glomerulosclerosis and CKD progression^39,46,49^.

#### Molecular changes

Podocyte structure and function depend on the highly organized molecular arrangement of pedicels in their various domains, especially in the filtration and cell signalling compartments^50^. Thus, molecular changes could negatively impact the function and viability of podocytes, leading to pathological consequences such as proteinuria and loss of renal function^46^.

Proteinuria is an early manifestation of podocyte dysfunction and also a typical sign of glomerulopathies^47^. In general, it reflects the existence of disorders affecting structural proteins of the slit diaphragm, adhesion molecules or actin cytoskeleton, generating GFB dysfunction^50^.

**Figure 3:**
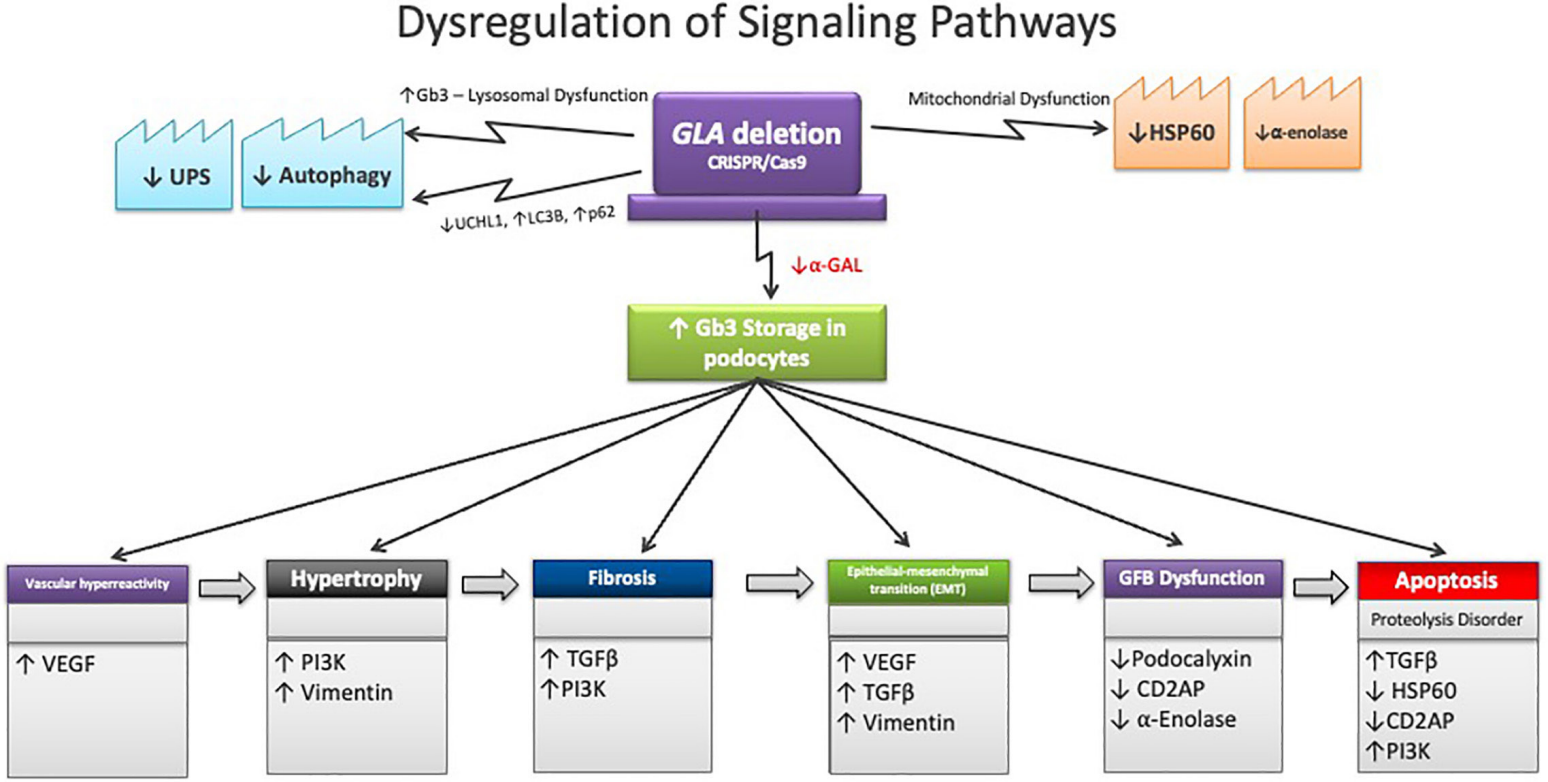
Scheme integrating the deregulated signaling pathways underlying the pathogenic effects on biological processes of in vitro podocytes subjected to prior GLA gene deletion (CRISPR/Cas9) expressing Fabry disease phenotype.

Glycosphingolipids, whose metabolism is altered by α-Gal deficiency, are key components of cell membrane structures called lipid “raft”, in which receptors and molecules involved in cell signaling of the slit diaphragm are interconnected. These are essential for the proper structuring and functioning of podocytes^51^. In FD, the significant accumulation of unprocessed glycosphingolipid substrates and the associated lysosomal dysfunction promote deregulation of signaling pathways crucial for podocyte functional performance and viability (Figure 3).

## Discussion

Renal involvement represents one of the primary morbidities with adverse impact on the prognosis of FD patients. While the severity and clinical outcomes of this nephropathy are evident, it should be considered that renal manifestations, including proteinuria and progressive CKD, are nonspecific, and present late as a consequence of early-onset podocyte injury^30,33,52^. Podocyte injury and loss are central events in the development of FD nephropathy, and explaining their mechanisms is crucial. Such elucidation may contribute to the understanding of more prevalent nephropathies that use similar pathogenic pathways.

Gene disruption techniques have brought innovative models and valuable insights to the study of pathophysiological mechanisms. From this perspective, in a model developed by our group, the FD phenotype was obtained in immortalized human podocytes in culture through *GLA* gene deletion using CRISPR/Cas9 technology^53^. In this research, multiple molecular alterations were observed, such as changes in the composition of intracellular proteins (UCHL1, HSP60 and α-enolase) involved in important biological processes, and deregulated signaling pathways, with increased expression of TGF-β, VEGF, vimentin and PI3K, as well as podocalyxin and CD2AP downregulation^53,54^. Podocytes lacking α-Gal enzyme activity came to express a profile of responses related to hypertrophy, fibrosis, vascular hyperreactivity and epithelial-mesenchymal transition. In addition, defects in autophagy (LC3B and p62 overexpressed) and greater apoptosis were confirmed^52,54^. The underexpression of podocyte structural proteins (podocalyxin and CD2AP) is associated with several deleterious effects, such as loss of GFB selectivity, pedicel effacement, slit diaphragm decomposition and actin cytoskeleton disruption (Figure 3).

The mechanisms by which substrates (Gb3) not degraded within lysosomes lead to cell dysfunction in FD are still poorly understood^30,55^. Pathological changes likely result, in part, from functional disorders of lysosomes, catabolic compartments of the cell that store pathogenic deposits.

Autophagy, dependent on the lysosome, is an essential process for the preservation of long-lived, differentiated cells such as podocytes, by which cells degrade and recycle proteins to maintain their homeostasis and integrity^56,57^. Under baseline conditions, podocytes already demonstrate a high level of autophagy, which is intensified in adaptive responses to injury^58^. Proteolytic pathways collectively possess cytoprotective properties, as they regulate fundamental physiological processes in podocytes, such as slit diaphragm function, signaling pathway activity, actin cytoskeleton synthesis, cell differentiation and metabolism^55,59^. The failure of these processes increases the level of cytotoxic components and dysfunctional cytoplasmic proteins, leading to disruption of podocyte stability and outcomes such as proteinuria and kidney failure^57,60^.

A growing body of evidence highlights the loss of intracellular protein homeostasis associated with protein catabolism pathways, such as autophagy and the ubiquitin-proteasome system (UPS), acting in the pathogenesis of podocyte injury^57,59,61^. Autophagy disruptions have already been well documented in FD: Chévrier et al.^62^ also demonstrated autophagic pathway failure in renal cells and fibroblasts from FD patients; Liebau et al.^63^ showed that Gb3 accumulation in podocytes is associated with increased autophagosomes, suggesting that autophagy blockage plays a role in the pathogenesis of glomerular injury. Recent evidence reveals the concurrent impairment of UPS-associated autophagy in podocytes in an experimental model of FD^54^. This joint deregulation of proteolytic pathways in podocytes, highly dependent on protein homeostasis to maintain their complex structure and functionality, seems to contribute to exacerbating podocyte injury in FD^54^.

Podocyte dedifferentiation with loss of the epithelial phenotype associated with the acquisition of mesenchymal characteristics, known as epithelial-mesenchymal transition (EMT), has been implicated in the pathogenesis of podocytopathies^54,64^. In this process, there is a loss of apical-basal polarity, while intercellular junctions are decomposed, resulting in adhesion failures. These aspects are detrimental to podocyte function and viability, facilitating their detachment from the glomerular capillary^64^. The etiology of EMT is complex and involves deregulated signaling pathways leading to fibrosis and glomerulosclerosis, with TGF-β (transforming growth factor β) being an important inducer, resulting in irreversible kidney injury^64,65^.

**Figure 4:**
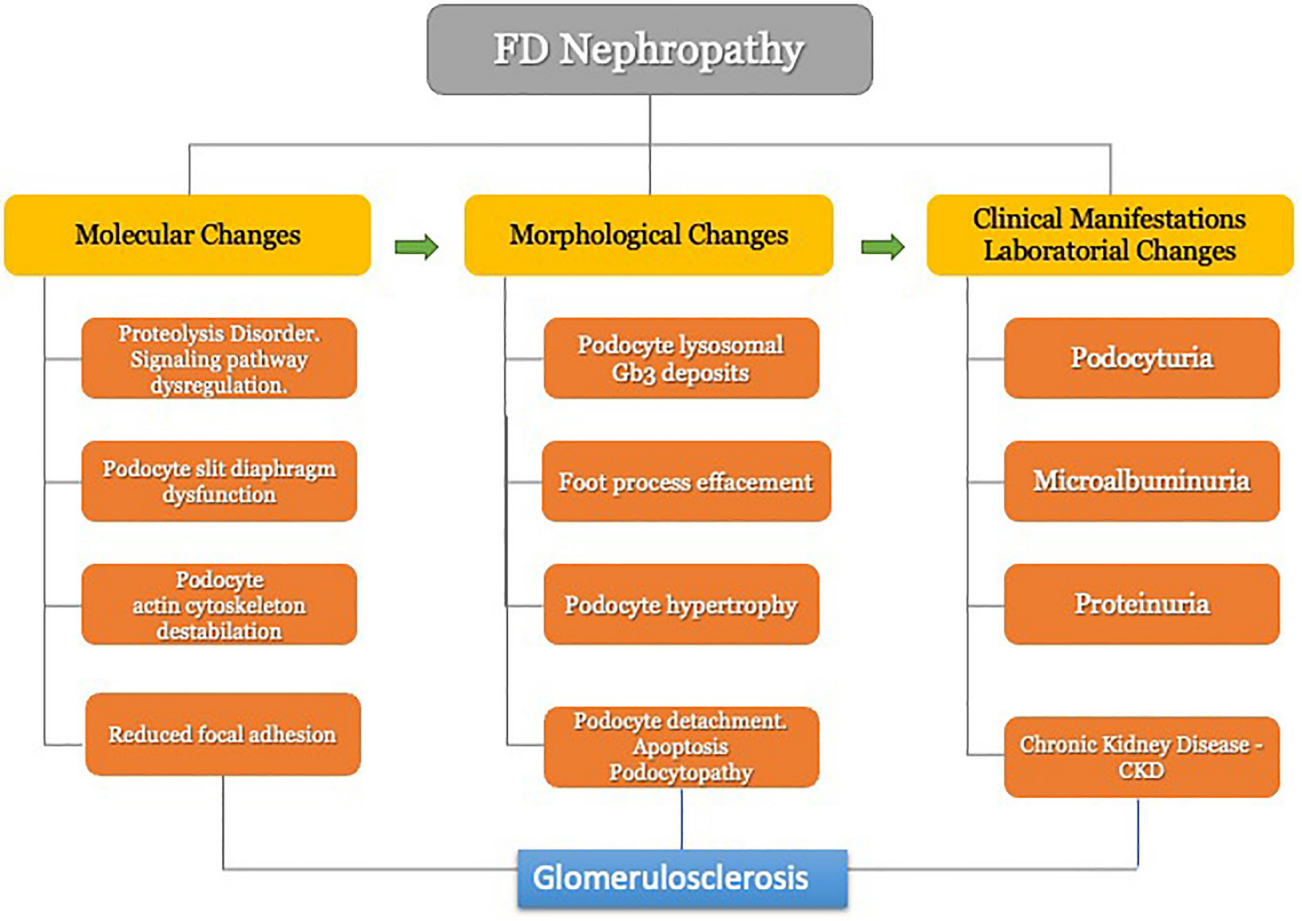
Sequence of molecular and cellular events implicated in the pathogenesis and progression of Fabry disease nephropathy.

In summary, FD nephropathy involves a series of structural and functional alterations, revealing a complex scenario in which podocyte injury and its consequences prevail. Podocytes undergo initial molecular modifications, preceding the morphological repercussions and subsequent clinical outcomes that culminate in progressive proteinuria and definitive loss of renal function (Figure 4)^8,54,55,66^. Cellular changes resulting from podocyte injury stem from a complex loss of protein homeostasis that masterfully regulates the physiology and structure of podocytes.

## Conclusion

FD nephropathy combines features of both hereditary and metabolic podocytopathy, wherein a complex network of molecular and cellular events is involved in its pathogenesis. The morbid consequences cannot be solely attributed to the simple deposition of unprocessed substrates within lysosomes, stemming from the genetic defect and impaired glycosphingolipid metabolism, but seem to involve cellular disorders that extend beyond the domains of the lysosome. In addition to deposits, molecular disturbances and deregulation of several cellular processes, including those related to lysosomes, play a significant role, preceding the podocyte injury that triggers clinical outcomes, ultimately leading to definitive loss of kidney function.

Understanding and controlling these mechanisms is paramount for the development of therapeutic strategies focused on podocyte protection, aimed not only at reducing substrate accumulation, but above all at correcting the associated molecular and cellular alterations.
