# Supplementary material for: The role of podocyte injury in the pathogenesis of Fabry disease nephropathy
Source: J Bras Nefrol. 2024 Jul 19;46(3):e20240035. doi: 10.1590/2175-8239-JBN-2024-0035en (PMC11287863; doi:10.1590/2175-8239-JBN-2024-0035en)
Supplement: Translation

# Translation: O papel da injúria podocitária na patogênese da nefropatia da doença de Fabry

**DOI:** 10.1590/2175-8239-JBN-2024-0035pt

**Authors:** José Tiburcio do Monte, Gianna Mastroianni Kirsztajn

**Affiliations:** 1 Universidade Federal do Piauí, Departamento de Clínica Geral, Teresina, PI, Brasil.; 2 Universidade Federal de São Paulo, Departamento de Medicina, São Paulo, SP, Brasil.

**Keywords:** Doença de Fabry, Podócito, Barreira de Filtração Glomerular, Autofagia, Insuficiência Renal Crônica

## Abstract

O acometimento renal é uma das mais severas morbidades da doença de Fabry (DF), enfermidade multissistêmica de depósito lisossômico com padrão de herança ligada ao cromossomo *X*, decorrente de variantes patogênicas do gene *GLA* (Xq22.2), que codifica a produção de alfa-galactosidase A (α-Gal), responsável pelo metabolismo de glicoesfingolipídeos. A atividade insuficiente dessa enzima lisossômica gera depósitos de substratos intermediários não processados, especialmente do globotriaosilceramida (Gb3) e derivados, desencadeando injúria celular e, posteriormente, disfunção de múltiplos órgãos, incluindo a nefropatia crônica. A lesão renal na DF é classicamente atribuída aos depósitos de Gb3 nas células renais, sendo os podócitos o alvo principal do processo patológico, nos quais as alterações estruturais e funcionais são instaladas de forma precoce e severa, configurando uma podocitopatia metabólica hereditária típica, cujas manifestações clínicas são proteinúria e falência renal progressiva. Embora os desfechos clínicos tardios e as alterações morfológicas estejam bem estabelecidos nessa nefropatia, os mecanismos moleculares que deflagram e aceleram a injúria podocitária ainda não estão completamente elucidados. Podócitos são células altamente especializadas e diferenciadas que revestem a superfície externa dos capilares glomerulares, desempenhando papel essencial na preservação da estrutura e função da barreira de filtração glomerular, sendo alvos frequentes de injúria em muitas nefropatias. A disfunção e depleção dos podócitos glomerulares são, além disso, eventos cruciais implicados na patogênese da progressão da doença renal crônica. Revisaremos a biologia dos podócitos e seu papel na regulação da barreira de filtração glomerular, analisando as principais vias patogênicas envolvidas na lesão podocitária, especialmente relacionadas à nefropatia da DF.

## Introdução

A doença de Fabry (DF) é uma desordem genética de depósito lisossômico com padrão de transmissão ligada ao cromossomo *X*, cuja incidência é de aproximadamente 1 em 40-60.000 homens nascidos vivos^1^. A enfermidade é decorrente de variantes patogênicas do gene *GLA* (localizado na região Xq22.2), responsável pela transcrição da enzima alfa-galactosidase A (α-Gal), que atua no catabolismo de glicoesfingolipídeos. A falha de atividade dessa enzima produz acúmulo progressivo no interior dos lisossomos de substratos intermediários tóxicos, como o globotriaosilceramida (Gb3), deflagrando um lento processo de injúria celular que posteriormente se manifesta com disfunção em órgãos alvos como coração, sistema nervoso e rins^2^.

Até o momento, mais de mil mutações do gene *GLA* foram descritas, correlacionadas ao quadro heterogêneo e multissistêmico da DF, reunindo um amplo espectro de manifestações clínicas, que inclui o fenótipo clássico em homens e a enfermidade de início tardio. As mulheres afetadas, como são heterozigotas (XX), costumam apresentar manifestações clínicas atenuadas devido ao fenômeno de inativação aleatória de um dos cromossomos X na fase embrionária^3^.

O envolvimento renal é a principal causa de morbimortalidade na DF. A nefropatia nessa desordem representa uma podocitopatia de origem genética e metabólica, visto que a lesão podocitária desempenha um papel central na patogênese^4–6^. No contexto geral, a injúria podocitária e suas consequências são eventos presentes na maioria das nefropatias proteinúricas e estão, além disso, implicadas na progressão da doença renal crônica (DRC) de diversas etiologias^7^.

A nefropatia da DF é complexa e envolve múltiplos aspectos moleculares e alterações estruturais que antecedem os eventos clínicos^8–10^. O desarranjo metabólico induzido pela inativação de gene *GLA* parece promover a produção de mediadores secundários que culminam com a injúria podocitária, desencadeando a nefropatia crônica progressiva que resulta em glomeruloesclerose e falência renal. Torna-se necessário aprofundar o conhecimento dos mecanismos moleculares que integram a nefropatia da DF a partir do defeito genético, indo além dos depósitos lisossômicos de Gb3^8^. O armazenamento cumulativo de substratos, causado pela deficiência enzimática, pode destacar apenas um aspecto da patogênese da doença, enquanto os mecanismos moleculares envolvidos só serão completamente entendidos se considerarmos as funções celulares afetadas^9,11^.

Nesta revisão, exploraremos a biologia dos podócitos e o seu papel na regulação da barreira de filtração glomerular, investigando a sequência de eventos moleculares e celulares implicados na patogênese e progressão da nefropatia da DF. Embora rara, a abordagem dessa desordem genética pode contribuir para a compreensão dos processos patológicos compartilhados pelas nefropatias mais prevalentes.

### Glomérulo e Barreira de Filtração Glomerular

A filtração do sangue realizada no córtex renal pelos glomérulos é essencial para a regulação do volume e da composição do organismo, manutenção da homeostase e desempenho integral da função renal. Os glomérulos formam uma estrutura esférica delimitada pela cápsula de Bowman, constituída por uma rede de capilares enovelados, de alta permeabilidade, submetidos a elevada pressão hidrostática, estabelecida pela diferença de resistência entre as arteríolas eferentes e aferentes, responsáveis pela saída e entrada do sangue no glomérulo, respectivamente^12^.

O ultrafiltrado glomerular do plasma é a maior transferência de fluidos do organismo, cujo volume atinge aproximadamente 180 litros por dia. Nesse processo, continuamente executado pelos glomérulos saudáveis, no ritmo de 100–125 mL/min/1,73 m^2^ de superfície corpórea, é necessário conservar no interior dos capilares os componentes essenciais do sangue: células, nutrientes e proteínas de alto peso molecular, como a albumina, enquanto eletrólitos, toxinas urêmicas, água, glicose e pequenos solutos são lançados no espaço urinário da cápsula de Bowman para serem processados pelos túbulos renais, na região medular, para finalmente formar a urina^13^.

A baixa excreção urinária de albumina (que fisiologicamente não ultrapassa 30 mg na urina de 24 horas) diante da sua elevada concentração no sangue (superior a 4000 mg em 100 mL de plasma) dimensiona a propriedade altamente seletiva do glomérulo (superior a 99,99%) na conservação dessa proteína^14^.

O filtro glomerular está centralizado na barreira de filtração glomerular (BFG), estrutura que reúne três camadas paralelas, e é assim constituída: (i) internamente, pelo endotélio fenestrado, (ii) no centro, pela membrana basal glomerular (MBG) e, (iii) externamente, pela camada de células epiteliais viscerais ou podócitos^15^. A MBG, por sua vez, é formada pela fusão da matriz extracelular produzida pelo endotélio subjacente e pelos podócitos sobrejacentes, estruturando uma rede cujo constituinte principal é o colágeno do tipo IV, ao qual ligam-se glicoproteínas (heparan sulfato, laminina e proteoglicanos) que expressam carga elétrica negativa, conferindo-lhe propriedade eletrostática, aspecto que colabora para impedir a filtração de proteínas de carga aniônica como a albumina^16^.

A BFG atua, portanto, como um filtro seletivo, de alta eficiência, expressando barreiras mecânica e eletrostática, que simultaneamente impedem a passagem de moléculas com base no tamanho, forma e carga, produzindo o ultrafiltrado em fluxo e composição do qual depende o desempenho funcional do rim^16^.

### Estrutura e Função dos Podócitos

A camada externa da BFG é estrategicamente constituída pelos podócitos, células epiteliais viscerais que envolvem os capilares glomerulares a partir do polo vascular, delimitando a fronteira entre as estruturas vasculares e o espaço urinário^17^. O glomérulo saudável possui aproximadamente 500 a 600 podócitos, representando 30% dos seus componentes celulares^18^.

Os podócitos são as células mais diferenciadas e especializadas do rim^19^. Têm corpo celular volumoso que flutua no espaço urinário, do qual emerge um extenso citoplasma arborizado que se projeta em direção aos capilares formando os processos primários, secundários e os pedicelos (processos podálicos)^17^. Os pedicelos cobrem toda a extensão da MBG formando interdigitações, entre si e com os pedicelos dos podócitos adjacentes, através de junções intercelulares especializadas denominadas de diafragma da fenda.

A complexa arquitetura molecular dos podócitos reúne diversas proteínas interligadas, agrupadas em três domínios principais nos pedicelos: basal, apical e juncional (Figura 1)^17^. O domínio basal representa a superfície de contato aderida à MBG, contendo vários tipos de integrinas e distroglicanos, sendo a integrina α3β1 a mais abundante e a principal responsável pela adesão focal do podócito na MBG, impedindo o seu desprendimento do glomérulo. O domínio apical, voltado para o espaço urinário, possui uma superfície de carga aniônica, conferida pela presença de proteínas como a podocalixina, que, além de reforçar a barreira eletrostática da BFG, dificultando o escape de albumina, evita a adesão do podócito à cápsula de Bowman, mantendo os pedicelos adjacentes separados. O domínio juncional abrange o diafragma da fenda, cujo padrão de interdigitação semelhante a um zíper estabelece a última barreira ultrapassada pelo filtrado glomerular (Figura 1)^20^.

**Figura 1:** Esquema representando os pedicelos dos podócitos interligados no diafragma da fenda aderidos sobre a MBG e o endotélio subjacente, formando a BFG. Destaque para as principais proteínas estruturais nos seus domínios correspondentes: nefrina (domínio juncional do diafragma da fenda), podocalixina (domínio apical) e integrinas α3β1 (domínio basal), articuladas ao citoesqueleto de actina.

O diafragma da fenda simboliza a unidade funcional do podócito por exercer a mais estreita e definitiva barreira responsável pela permeabilidade seletiva do glomérulo^20,21^. Essa estrutura é preenchida por proteínas transmembranas específicas como a nefrina, principal componente dessa junção intercelular especializada, essencial para a organização e manutenção da integridade dos podócitos (Figura 1)^22^. Microfilamentos de actina formam o citoesqueleto dos pedicelos, fornecendo o suporte estrutural, além de permitir a contração e o relaxamento dos podócitos, regulando a superfície de filtração do capilar glomerular. Outras proteínas são essenciais, como a proteína adaptadora CD2AP (CD2-associated protein) e a podocina, atuam como âncoras ligadas à nefrina, fazendo a conexão do diafragma da fenda ao citoesqueleto de actina (Figura 1). O complexo proteico do diafragma da fenda e a interação da nefrina-CD2AP são essenciais para que a permeabilidade seletiva e a ultrafiltração do plasma sejam rigorosamente executadas pela BFG^22,23^.

Deste modo, a arquitetura molecular dos pedicelos configura uma rede complexa de interações entre diferentes proteínas e moléculas de sinalização, aspecto que garante a comunicação precisa entre os diferentes compartimentos celulares com o meio adjacente através das proteínas transmembranas específicas, que atuam como receptores interligados ao citoesqueleto de actina, modulando a forma e a função dos podócitos, emitindo respostas rápidas e eficazes às mudanças do ambiente glomerular^21^.

Além do controle da superfície e da permeabilidade da BFG, os podócitos têm outras funções, como a síntese e o reparo de componentes da MBG, produção de substâncias parácrinas que atuam como fator de crescimento sobre as células endoteliais e mesangiais, tais como o VEGF (fator de crescimento do endotélio vascular) e o PDGF (fator de crescimento derivado de plaquetas), promovendo a comunicação intraglomerular através de múltiplas vias de sinalização. Os podócitos realizam ainda a remoção de proteínas e imunoglobulinas não filtradas por endocitose, evitando a obstrução da membrana filtrante e, também, interagem com o sistema imune, atuando como células apresentadoras de antígenos ou como receptores de componentes do sistema do complemento e de imunoglobulinas, podendo ser alvo de agressões imunomediadas em algumas glomerulopatias^24^.

Compreende-se, dessa forma, que a função renal e a integridade da BFG são completamente dependentes da quantidade e do funcionamento adequado dos podócitos. O conjunto desse extraordinário trabalho, abrangendo a conexão e o monitoramento das outras células glomerulares, coloca o podócito na posição de comando do glomérulo.

## Nefropatia da Doença de Fabry

O comprometimento renal configura uma causa importante de morbimortalidade na DF^25,26^. No fenótipo clássico da enfermidade, a maioria dos homens portadores de mutações *nonsense* desenvolve microalbuminúria na infância e adolescência, evoluindo para proteinúria clínica a partir da juventude. Entre a terceira e a quarta década de vida, tem início a progressão da doença renal crônica (DRC), que atinge o estágio mais avançado por volta da quinta década de vida, requerendo introdução de terapia renal substitutiva (diálise ou transplante) (Figura 2)^6,25,27,28^. Nas mulheres e nos portadores de variantes de início tardio, o comprometimento renal costuma ser menos severo e ter evolução mais branda.

**Figura 2:** Resumo da evolução da nefropatia da doença de Fabry em homens portadores do fenótipo clássico da enfermidade, correlacionando o quadro clínico e laboratorial com as alterações morfológicas correspondentes.

Do ponto de vista clínico, o envolvimento renal da DF é caracterizado pela proteinúria de intensidade crescente e pelo declínio gradual da função renal. A proteinúria pode surgir na infância ou adolescência, refletindo a primeira manifestação clínica dessa nefropatia^29^. Entretanto, análises de biópsias renais revelaram a existência de alterações morfológicas ultraestruturais proeminentes nos podócitos antes de os sinais clínicos da doença renal tornarem-se evidentes^30^. A histopatologia renal da DF destaca podócitos hipertróficos com vacuolização citoplasmática de aparência espumosa e corpos de inclusão de aspecto multilamelar, descritos como figuras de mielina ou corpos zebroides, correspondentes aos depósitos de Gb3 acumulados nos lisossomos^31^.

Na história natural do fenótipo clássico da nefropatia da DF, os depósitos de Gb3 já estão presentes precocemente nos podócitos, iniciando-se na vida intrauterina^32^. Com o tempo e o aumento da idade do paciente, maior proporção do citoplasma é ocupada pelos depósitos não processados, prejudicando a funcionalidade^30,33^. A fase subclínica, observada na infância e adolescência, caracterizada por microalbuminúria, corresponde às alterações morfológicas do tipo apagamento dos pedicelos e hipertrofia dos podócitos. Na etapa seguinte, a piora dos parâmetros morfológicos dos podócitos se associa ao aumento da proteinúria e à piora da função renal, instalando-se a fase clínica dessa nefropatia (Figura 2)^34^. A seguir, ocorre uma rápida progressão da DRC, reflexo da depleção acelerada do número de podócitos (podocitopenia) e do desenvolvimento de glomeruloesclerose, intensificada a partir dos 30 anos de idade (Figura 2)^27,28,33^. A severidade da lesão podocitária encontra-se, dessa forma, fortemente associada com a progressão da nefropatia na DF (Figura 2).

Nesse contexto, os exames de função renal utilizados na rotina clínica que identificam proteinúria e taxa de filtração glomerular reduzida já revelam a lesão renal avançada^35^. Em um estudo prévio, identificamos uma correlação positiva entre a podocitúria e a excreção urinária de albumina em pacientes com DF^36^. Entretanto, a podocitúria, que poderia exercer o papel de biomarcador precoce do envolvimento renal, ocorre de forma descontínua e variável e não está disponível na rotina laboratorial.

Esses dados reforçam a necessidade de pesquisa de novos biomarcadores da injúria podocitária que possibilitem identificar a fase da nefropatia anterior ao surgimento das alterações estruturais e funcionais irreversíveis, otimizando, dessa forma, a indicação e eficácia de tratamentos específicos, como a terapia de reposição enzimática ou chaperonas farmacológicas (migalastat)^5,33,37^.

### Fisiopatologia da Injúria do Podócito na Nefropatia da Doença de Fabry

A injúria do podócito é o fator determinante para o desenvolvimento e a progressão da nefropatia da DF. Os depósitos lisossômicos de Gb3 configuram a primeira etapa das complexas vias patológicas que resultam na injúria podocitária nessa enfermidade^8,30^.

Uma vez que os podócitos exibem limitada capacidade regenerativa, própria de células pós-mitóticas, a lesão e a perda de podócitos, seja por apoptose ou destacamento, são eventos irreversíveis. Os podócitos são lesados tanto a nível molecular quanto morfológico no processo patológico da DF.

#### Alterações morfológicas

Pelo fato de terem atingido um alto estágio de diferenciação e especialização, próprio de células pós-mitóticas, os podócitos perderam a capacidade de se dividir, aspecto que os torna especialmente vulneráveis aos diversos insultos^38^. Os podócitos raramente sofrem mitose e, quando entram no ciclo celular, não completam a citocinese, surgindo mitoses catastróficas (aberrantes), na qual a divisão celular defeituosa produz células poliploides ou polinucleadas, sem aderência à MBG^39^.

Em resposta à injúria, surgem alterações morfológicas adaptativas dos podócitos, tais como: apagamento dos pedicelos, hipertrofia e destacamento. A severidade dessas respostas depende especialmente da intensidade e duração do insulto, que na DF ocorre de forma contínua pelo estoque cumulativo dos depósitos patogênicos que se intensificam com o avançar da idade do paciente.

**1) Fusão e apagamento dos pedicelos**

O apagamento dos pedicelos é caracterizado pela perda das interdigitações típicas, resultado da fusão e do desaparecimento do diafragma da fenda, com achatamento da camada podocitária^39,40^. Esse processo é desencadeado pela desestruturação do citoesqueleto de actina, tendo sido visualizado pela primeira vez na década de 1950, com a implantação da microscopia eletrônica, sendo esse achado associado às glomerulopatias proteinúricas, independentemente da etiologia^40^.

A lesão dos podócitos, pelo acúmulo progressivo de Gb3, está associada ao apagamento dos pedicelos. Essa alteração morfológica é considerada um marcador precoce da nefropatia em desenvolvimento e foi descrita em crianças a partir de 10 anos com o fenótipo clássico da DF que ainda não apresentavam evidências clínicas de comprometimento renal (Figura 2)^10,32,41^. A densidade das inclusões de Gb3 nos podócitos e a fusão dos pedicelos se acentuam com a idade do paciente, correlacionando-se com o aumento da proteinúria^30,33^.

**2) Destacamento dos podócitos e podocitúria**

A injúria crônica e persistente dos podócitos levam ao desprendimento destes do glomérulo, seja pelo aumento da tensão mecânica e/ou pela falha da adesão à MBG^42^. A pressão hidrostática, embora seja necessária para a filtração, gera estresse hemodinâmico sobre os podócitos quando se eleva além dos níveis fisiológicos. O estresse biomecânico excessivo causado pela hiperfiltração glomerular, como ocorre na hiperatividade do sistema renina-angiotensina, aumenta a tensão de cisalhamento exercida pelo ultrafiltrado glomerular nos pedicelos, opondo-se à fixação deles no capilar glomerular^43,44^. A adesão à MBG prejudicada desempenha um papel importante sobretudo em enfermidades genéticas ou adquiridas que envolvam alterações moleculares das proteínas estruturais do diafragma da fenda, levando ao surgimento de expressiva podocitúria^20^. A intensidade da podocitúria, além disso, tem sido correlacionada com a severidade da nefropatia na DF^42^.

**3) Hipertrofia dos podócitos**

Na DF, os podócitos apresentam aumento de volume e hipertrofia pela deposição contínua e cumulativa do Gb3^33^. Além disso, a redução da quantidade de podócitos por glomérulo, pelo destacamento excessivo, estimula o remodelamento das células remanescentes para cobrir as lacunas da cobertura podocitária da MBG^39,45,46^. O volume glomerular aumenta de forma acelerada com a idade, enquanto o número de podócitos diminui, resultando em uma densidade de podócitos reduzida^33^. A hipertrofia compensatória dos podócitos enfraquece sua adesão à MBG, tornando-os mais vulneráveis à lesão, agravando a disfunção e acelerando a progressão da doença renal crônica^39,47,48^.

Adicionalmente, se a hipertrofia compensatória não consegue superar a perda dos podócitos, surgem sinéquias e ocorre colapso de alças capilares, desencadeando o processo de esclerose glomerular^7^.

Enquanto as alterações do tipo apagamento dos pedicelos ou hipertrofia são potencialmente reversíveis, a perda de podócitos, por destacamento ou apoptose, caracteriza um evento irreversível^39^. Nesse estágio, a lesão podocitária leva à redução do número e densidade dessas células no glomérulo, culminando no processo irreversível de glomeruloesclerose e progressão da DRC^39,46,49^.

#### Alterações moleculares

A forma e a função podocitária dependem do arranjo molecular altamente organizado dos pedicelos em seus diversos domínios, especialmente nos compartimentos de filtração e sinalização celular^50^. Sendo assim, alterações moleculares podem provocar impacto negativo na função e na viabilidade dos podócitos, levando a consequências patológicas, como proteinúria e perda da função renal^46^.

A proteinúria é uma manifestação precoce da disfunção podocitária e, também, um sinal típico das glomerulopatias^47^. Em geral, reflete a existência de distúrbios que afetam as proteínas estruturais do diafragma da fenda, das moléculas de adesão ou do citoesqueleto de actina, gerando disfunção da BFG^50^.

Os glicoesfingolipídios, cujo metabolismo está alterado pela deficiência da α-Gal, são os principais componentes de estruturas da membrana celular denominadas “jangadas” lipídicas, nas quais estão interligados os receptores e as moléculas envolvidas na sinalização celular do diafragma da fenda, essenciais para a estruturação e o funcionamento adequados dos podócitos^51^. Na DF, o acúmulo expressivo de substratos não processados dos glicoesfingolípideos e a disfunção lisossômica associada promovem desregulação das vias de sinalização que são essenciais para o desempenho funcional e a viabilidade dos podócitos (Figura 3).

**Figura 3:** Esquema integrando as vias de sinalização desreguladas subjacentes aos efeitos patogênicos nos processos biológicos de podócitos *in vitro* submetidos à deleção prévia do gene *GLA* (CRISPR/Cas9) expressando o fenótipo da doença de Fabry.

## Discussão

O envolvimento renal representa uma das principais morbidades com impacto adverso no prognóstico de pacientes com DF. Embora a gravidade e os desfechos clínicos dessa nefropatia sejam evidentes, deve-se considerar que as manifestações renais, que incluem proteinúria e DRC progressiva, são inespecíficas e surgem tardiamente como consequência da injúria podocitária precocemente instalada^30,33,52^. A lesão e a perda de podócitos são os eventos centrais para o desenvolvimento da nefropatia da DF, cujos mecanismos precisam ser melhor elucidados, pois podem contribuir para o entendimento de nefropatias mais prevalentes que utilizam vias patogênicas similares.

As técnicas de ruptura genética trouxeram modelos inovadores e informações valiosas para os estudos de mecanismos fisiopatológicos. Nessa perspectiva, em um modelo desenvolvido por nosso grupo, o fenótipo da DF foi obtido em podócitos humanos imortalizados em cultura, através da deleção do gene *GLA* utilizando a tecnologia CRISPR/Cas9^53^. Nesta pesquisa, foram observadas múltiplas alterações moleculares, tais como: mudanças da composição de proteínas intracelulares (UCHL1, HSP60 e α-enolase) que participam de processos biológicos importantes e vias de sinalização desreguladas, com aumento da expressão do TGF-β, VEGF, vimentina e PI3K, além de regulação negativa da podocalixina e CD2AP^53,54^. Os podócitos sem atividade da enzima α-Gal passaram a expressar um perfil de respostas relacionadas à hipertrofia, fibrose, hiper-reatividade vascular e transição epitélio-mesenquimal, além de comprovar defeitos da autofagia (LC3B e p62 superexpressos) e maior apoptose^52,54^. A subexpressão das proteínas estruturais do podócito (podocalixina e CD2AP) se associa a diversos efeitos deletérios, tais como: perda da seletividade da BFG, apagamento dos pedicelos, decomposição do diafragma da fenda e desestruturação do citoesqueleto de actina (Figura 3).

Os mecanismos pelos quais os substratos (Gb3) não degradados nos lisossomos levam à disfunção celular na DF são ainda pouco compreendidos^30,55^. As alterações patológicas provavelmente resultam, em parte, dos distúrbios funcionais dos lisossomos, compartimentos catabólicos da célula que estocam os depósitos patogênicos.

A autofagia, dependente do lisossomo, é um processo essencial para a preservação de células diferenciadas e de vida longa, como os podócitos, pelo qual as células degradam e reciclam proteínas para manter a sua homeostase e integridade^56,57^. Em condições basais, os podócitos já demonstram alto nível de autofagia, intensificado nas respostas adaptativas à injúria^58^. As vias proteolíticas possuem coletivamente propriedades citoprotetoras, visto que regulam processos fisiológicos fundamentais dos podócitos, tais como a função do diafragma da fenda, atividade das vias de sinalização, síntese do citoesqueleto de actina, diferenciação e metabolismo celular^55,59^. A falha desses processos aumenta o nível de componentes citotóxicos e proteínas citoplasmáticas disfuncionais, levando à ruptura da estabilidade dos podócitos e desfechos como proteinúria e insuficiência renal^57,60^.

Um conjunto crescente de evidências destaca a perda da homeostase proteica intracelular associadas às vias do catabolismo proteico, como a autofagia e o sistema ubiquitina-proteasoma (SUP), atuando na patogênese da injúria podocitária^57,59,61^. Interrupções da autofagia já foram bem documentadas na DF: Chévrier et al.^62^ também demonstraram falha da via autofágica em células renais e fibroblastos de pacientes com DF; Liebau et al.^63^ demonstraram que o acúmulo de Gb3 em podócitos está associado a um aumento dos autofagossomos, sugerindo que o bloqueio da autofagia desempenha um papel na patogênese da lesão glomerular. Evidência recente revela a deficiência concomitante da autofagia associada ao SUP em podócitos em um modelo experimental da DF^54^. Essa desregulação conjunta das vias proteolíticas em podócitos, extremamente dependente da homeostase proteica para manter a sua complexa estrutura e funcionalidade, parece contribuir para exacerbar a lesão podocitária na DF^54^.

A desdiferenciação dos podócitos com a perda do fenótipo epitelial associado à aquisição de características mesenquimais, denominada transição epitélio mesenquimal (TEM), tem sido implicada na patogênese das podocitopatias^54,64^. Nesse processo, ocorre perda da polaridade apical-basal, enquanto as junções intercelulares são decompostas, resultando em falhas da adesão, aspectos prejudiciais à função e viabilidade dos podócitos, facilitando o seu destacamento do capilar glomerular^64^. A etiologia da TEM é complexa e envolve vias de sinalização desreguladas que levam à fibrose e glomeruloesclerose, na qual o TGF-β (fator de crescimento transformador β) é um importante indutor, resultando em lesão renal irreversível^64,65^.

Em suma, a nefropatia da DF envolve uma série de alterações estruturais e funcionais, revelando um cenário complexo em que predomina a injúria podocitária e suas consequências. Os podócitos passam por modificações moleculares iniciais, precedendo as repercussões morfológicas e os desfechos clínicos subsequentes que culminam com proteinúria progressiva e a perda definitiva da função renal (Figura 4)^8,54,55,66^. As alterações celulares decorrentes da injúria podocitária resultaram de uma complexa perda da homeostase das proteínas que magistralmente regulam a fisiologia e a estrutura dos podócitos.

**Figura 4:** Sequência de eventos moleculares e celulares implicados na patogênese e progressão da nefropatia da doença de Fabry.

## Conclusão

A nefropatia da DF reúne características de uma podocitopatia hereditária e metabólica, na qual uma complexa rede de eventos moleculares e celulares está envolvida na patogênese. As consequências mórbidas não podem ser atribuídas exclusivamente à simples deposição de substratos não processados nos lisossomos, a partir do defeito genético e da falha do metabolismo de glicoesfingolipídeos, mas parecem envolver distúrbios celulares que extrapolam os domínios do lisossomo. Além dos depósitos, distúrbios moleculares e a desregulação de vários processos celulares, incluindo os relacionados aos lisossomos, desempenham um papel significativo, antecedendo a lesão podocitária que deflagra os desfechos clínicos, culminando com a perda definitiva da função renal.

A compreensão e o controle desses mecanismos são cruciais para o desenvolvimento de estratégias terapêuticas focadas na proteção do podócito, visando não apenas à redução do acúmulo de substratos, mas sobretudo à correção das alterações moleculares e celulares associadas.
